# The importance of initial response during outbreaks: A perspective from observations on COVID-19

**DOI:** 10.1017/ice.2020.150

**Published:** 2020-04-20

**Authors:** Yao Yu Yeo, Bruce Ganem

**Affiliations:** 1 Department of Microbiology and Immunology, Cornell University, Ithaca, New York; 2 Department of Chemistry and Chemical Biology, Cornell University, Ithaca, New York

*To the Editor*—Pandemics have occurred throughout history and have generally led to catastrophic aftermaths across all levels of society. Notable examples include the black death in the 14th century and the 1918 Spanish flu; both names strike like a universal apocalyptic chorus.^1^ Although the understanding of microbes, the emergence of epidemiology, and rapid advancement of science and medicine over the 19th and 20th centuries have kept many old pathogens at bay and have led to an unprecedented quality of life,^2^ the modern world has already faced 2 dangerous new pandemics (2009 H1N1 influenza and COVID-19) in the 21st century.

These recent pandemics are of particular concern not only because the medical and scientific communities were already aware of (and quite familiar with) both families of viruses but also because we currently live in an era that boasts stellar healthcare quality compared to just a century ago. For these reasons, the ongoing COVID-19 pandemic merits closer examination as a case study for learning new approaches to restraining future outbreaks.

The COVID-19 pandemic originated in Wuhan, China, in December 2019.^3^ It began as an epidemic but has since spread to >150 countries and sovereignties.^4^ Although some countries and sovereignties (eg, Singapore, Hong Kong, and Taiwan) have managed to contain the spread of SARS-CoV-2, others (eg, Italy, Spain, and the United States) have experienced an unexpected number of COVID-19 cases and are struggling with overburdened healthcare facilities despite the fact that all 6 of these countries (except Spain) quickly implemented travel restrictions to mainland China within the first week^5^ and executed domestic lockdowns to restrict community spread. However, a major difference that helps explain the disparity in the severity of the pandemic is evident in these countries’ initial responses to COVID-19.

**Fig. 1.**
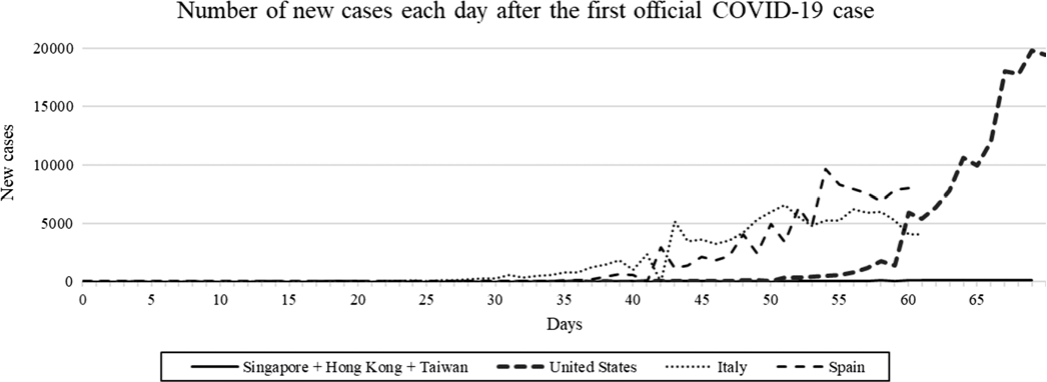
Number of new official COVID-19 cases in selected countries or sovereignties each day after the onset of the COVID-19 pandemic. By consensus, the actual number of cases in the United States, Italy, and Spain should be much higher, whereas Singapore, Hong Kong, and Taiwan have been reporting cases with accuracy and transparency. Notably, the sum of new cases across Singapore, Hong Kong, and Taiwan pale in comparison to those in the United States, Italy, and Spain. Raw data up to March 31, 2020, were obtained from official sources available online.

Three countries and sovereignties that have managed COVID-19 remarkably well (Singapore, Hong Kong, and Taiwan) happen to be densely populated regions whose citizens frequently travel to and from mainland China, which makes their achievement noteworthy. Since the emergence of SARS-CoV-2, these countries and sovereignties have made testing widely available and accessible and have carried out rigorous contact tracing, thus facilitating the diagnosis and treatment of every case. Ministers in these countries have also been warning about the impending outbreak and relaying accurate information around the clock. Although they initially created panic, these efforts resulted in a perpetual minimal relative number of new cases over the past few months (Fig. 1). Today, Singapore, Hong Kong, and Taiwan enjoy a reassuring sense of social calm and security.

By contrast, 3 countries that currently face sharp increases of new cases each day (ie, Italy, Spain, and the United States) either delayed or lacked sufficient access to reliable testing for various reasons (Fig. 1). One critical difference in their initial approaches was the overall sense of complacency by government officials, resulting in a reactive instead of proactive response. Italy, Spain, and the United States chose to falsely reassure the populace and to downplay the threat of COVID-19. Dissemination of coherent information about the pandemic only began after more than 1 month (in Italy)^6^ and 1.5 months (in Spain),^7^ upon observing a surge in new COVID-19 cases (Fig. 1). By that time, the pandemic had grown out of control in those countries and had overwhelmed healthcare facilities. The number of new cases in Italy has stabilized recently, suggesting that aggressive actions are working, but the crisis could have been averted had the country chosen to handle COVID-19 diligently from the start and ensured that sufficient COVID-19 testing was conducted sooner. As a result, Italy, Spain, and the United States are among the many countries experiencing severe social and economic turmoil today.

A central takeaway from the COVID-19 experience in these 6 countries is the strong association between initial action and disease severity. Countries that fared relatively well during the pandemic acted swiftly—from day 1—with testing, contact tracing, travel restrictions and lockdowns, and by circulating factual news, unlike those countries that delayed a rigorous response and rapidly fell behind in diagnosing and treating COVID-19 patients. The situation in the United States continues to unfold, and the outbreak has been characterized by conflicting and often unsubstantiated statements from government officials to the American public.

Many have questioned how the healthcare system of a prosperous country could be overwhelmed so drastically in this era of sophisticated medical treatment and care, most notably with acute shortages of hospital beds, respirators, and ventilators. The answer lies in 2 concluding observations that bear on the capacity of the modern healthcare system in developed countries. First, the number of hospital beds has fallen as the length of stay is reduced in response to pressures from lower insurance costs. Furthermore, increasing numbers of patients are receiving medical and surgical care in outpatient facilities to increase hospital efficiencies.^8^ Second, understaffing and reduced ICU occupancies over the years,^9^ in a bid to reduce hospital-acquired infections,^10^ have undermined the ability to provide critical care during mass-casualty incidents. Although these changes reflect the progress of healthcare and society, they have seemingly “backfired” during the COVID-19 pandemic. However, this is not the case because advanced health care (quality-wise) in the United States would have easily handled COVID-19 if officials had instead chosen to face COVID-19 with tenacity at its inception like its Asian counterparts, which it actually had much time to do (Fig. 1).

Hopefully, the world will learn from the COVID-19 pandemic and will be in a better position to face the next inevitable pandemic. Perhaps diligent initial responses to outbreaks are indispensable for preventing future outbreaks from exploding into pandemics.
